# Identifying Acuity Level-Based Adult Emergency Department Use Time Trends Across Demographic Characteristics

**DOI:** 10.7759/cureus.13225

**Published:** 2021-02-08

**Authors:** Swarna S Weerasinghe, Sam G Campbell

**Affiliations:** 1 Department of Community Health and Epidemiology, Dalhousie University, Halifax, CAN; 2 Department of Emergency Medicine, Dalhousie University, Halifax, CAN

**Keywords:** emergency department visit rates, time trends, demographic characteristics of ed visit trends, canadian triage acuity scale based emergency visits

## Abstract

Introduction

Canadian emergency departments (EDs) are struggling under the weight of increased use by a growing population of elderly patients; those who lack proper housing; and those who lack family physicians to provide primary care. The Canadian Foundation for Healthcare Improvement projected a possible ED service utilization increase in Canada at a rate of 40% over three decades. This calls for local-level information on the time trends to understand demographic and temporal variations in the different geographical locations in the country. This study sought to identify and quantify acuity level-based per capita ED visit annual time trends for the 10-year period of 2006-2015 (by age, gender, and housing status). The aim is to provide detailed information on the time trends for demographically targeted ED planning locally. The lengthy record of data allows examining the changing directions in different time segments.

Material and methods

Administrative data from the largest emergency department in Halifax (Nova Scotia, Canada) was analyzed. Per capita adult ED visit rates (EDVR) based on Canadian Triage Acuity Scale (CTAS), age, gender, and housing status were analyzed. Trends in the age-gender-based standardized rates using 2011 census city population data were also estimated in order to account for the population increase in the city.

Results

No study in Canada has documented the possibility of flattening the escalating ED visit trend by maintaining an annual declining trend in low-acuity-level visits or documented a threshold rate of decline to be maintained. This study observed that the annual linear per capita non-homeless EDVR increment trend (328/year, CI:245-411, per 100,000) for all-acuity-level visits - noted for a ten-year period - would become stable when low-acuity-level CTAS4-5 visit declining trends (427/year, CI:350-503 and 121/year, CI:79-163, per 100,000) - noted for the period of 2012-2015 - were maintained at the same magnitude and direction*.* Alarming annual emergent (high acuity level of CTAS2) EDVR increase equivalent to 335/year (CI:280-391, per 100,000) was noted for all combined visits, from all age, gender, and housing groups visits. The highest incremental rate noted among above-50-year-olds (521/year, per 100,000, 95% CI:433-608) was neither driven by overall increasing population census numbers nor by increasing aging population numbers. We found statistically similar age-gender standardized rates (294/year, CI: 207-382) for all ED visits and (316/year, CI:261-372) for CTAS2 level visits, when adjusted for annual population increase. Homeless ED visits did not contribute to the overall ED visit incremental trend. The highest annual homeless increment rate was shown for <30-year-old group high acuity CTAS-2 level visits (219/year, CI:193-246, per 100,000).

Conclusion

Neither the city population increase nor increased homeless visits contributed to ED visit annual per capita incremental trends in the city of Halifax. The increasing trend was chiefly driven by high-acuity-level visits by >50-year-old patients. Our findings suggest one way to make this escalating ED visit rates stable in the future is by maintaining the declining semi-urgent and non-urgent visit trends at the same rates estimated within the years 2012-2015. These findings highlight the potential directions for ED services planning to keep up with the growing demand for high-acuity-level ED services by the aging population.

## Introduction

Increased demand is placed on the Emergency Department (ED) service delivery partly due to the shortage of general practitioners to meet the demand of population growth in most Canadian cities. Across Canada, 15% of the population reported not having a family doctor [1]. This is often perceived as contributing to ED congestion and long wait times in ED, as people turn to the ED for problems that are more appropriately managed by family doctors. It is important to examine future needs and whether EDs need to adjust to (a) cope with a growing number of primary care patients, (b) those with purely demographic trends-related reasons for choosing the ED, and (c) growing needs of elderly patients with acute, time-sensitive illnesses.

Nova Scotia, where this study took place, has the third-highest ED use rate in Canada [2], with a notable shortage of family physicians, wherein up to 12.7% of the people are without a family doctor [3]. Based on media reports, even those with a family doctor must wait as long as six weeks for an appointment. The Canadian Foundation for Healthcare Improvement (CFHI) used 2013-2015 ED visit data and projected a possible ED service utilization increase in Canada at an alarming percentage of 40% from 15 million ED visits in 2013 to over 21 million by 2043 [4]. This ED visit projection was considered as mainly resulting from the aging population in Canada [4]. Canadian census data had confirmed an increase in the proportion of the population over the age of 60 years [5]. There is an urgent need to respond to the increased demand for ED services that matches these demographic trends and associated health risks when planning for future ED healthcare provision. Besides aging, the other two demographic characterizations were chosen based on the Canadian literature that suggest gender and sex differences in severity of presentations and patterns of ED use and compelling evidence from the Canadian literature confirming homeless people as having more acute and chronic health issues than the general population [6-7]. 

According to the Halifax Report on Homelessness, 47% of homeless people in Halifax in 2009 reported visiting the ED at least once during the previous 24 months [8]. Halifax homeless population is growing in numbers and it is important to understand the trends of acuity level based ED use of the homeless population. Providing timely ED care involves the management of input, throughput, and output, all which depends on the level of acuity of presentations. Canadian Triage Acuity Scale (CTAS) has been documented as a vital tool to use in situations when there is an increase in the volume of visits in high-acuity-level presentations [9]. There is a paucity of Canadian estimates on demographically based time trends of ED use across CTAS levels. 

## Materials and methods

The primary focus of this study is to examine CTAS-based, per capita ED Visit Rate (EDVR) and standardized ED visit rate (SEVR) time trends over a ten-year period across demographic groups determined by age, gender, and housing status. Other Canadian publications have presented the percentage of annual ED use time trends [6,10], but we have chosen to use per capita rates to account for demographic diversity-based population changes over time. By calculating rates of ED use across demographic characteristics in proportion to the same demographically categorized population numbers (from census data), accurate measure of trends and patterns reflecting population census changes can be obtained. Age-gender standardized rates provide rates after adjusting for population demographic changes over time.

To guide ED service planning, we estimated adult ED time trends (standardized and non-standardized rates) over a 10-year period, examining elements of demographic diversity in the study area such as housing status (homeless/non-homeless), age, and gender across the five levels of the Canadian Trauma Acuity Scale (CTAS) acuity levels. Understanding the demand that the population aging and increasing homelessness put on high-acuity-level care in comparison to the low-acuity-level care will equip ED care planners with much-needed estimates for resource allocations to provide equal access to all. 

Study design and setting

The study design was a retrospective cohort time-series study that explored ED visits over the time period 2006-2015. The study population comprised of all adults (above 16 years), who visited the Charles V. Keating Emergency and Trauma Centre at the Halifax Infirmary (HI), the largest emergency department in Halifax, Nova Scotia, Canada. Daily ED visits (outcome measure), broken down by data-driven age group categorization (<30, 30 to 50, and above 50 years, with equal percentage of ED visits in each group), gender, homeless/non-homeless and CTAS were extracted from the HI ED Information System (EDIS). Age cut-offs were chosen to have equal percentage of ED visits in each age group. Homelessness was captured in the EDIS as not having a fixed address or having an address of a shelter, verified using addresses of the homeless shelters in Halifax.

Data analysis

Likely data entry errors were excluded using filters of (a) wait time greater than 16 hours (two work shifts), (b) gender or visit date not recorded, and (c) age less than 16 years. We analyzed 64,6731, daily ED visits, over 3,652 days, categorized by age group, gender (male/female), housing status (homeless/non-homeless), and CTAS. Annual per capita EDVR for each demographic group was calculated by dividing the total annual group-specific ED visit counts for each CTAS by the same group specific annual total census of the Halifax municipality, retrieved from Statistics Canada [5]. Halifax point in time homeless counts were obtained from Halifax city Mobile Outreach Street Health program [11]. Confidence intervals were calculated using exact Poisson rates formula for EDVR and SEVR [12]. Standardized ED visit rates were calculated using 2011 Halifax municipality age and gender categorized census numbers, available from Statistics Canada [5] as the reference population. This standardization approach has been used to compare Canadian homeless mortality rates with Canadian low-income population mortality rates [6]. The study was approved by the Nova Scotia Health Authority ethics review board. The data were analyzed using R statistical analysis software (R Foundation for Statistical Computing, Vienna, Austria) [13]. All annual EDVR and SEVR rate estimates presented were extrapolated to a population of 100,000, all confidence intervals were based on 95% level of confidence, and statistical significance was assessed at an alpha level of significance of 0.05 by considering non-overlapping 95% confidence intervals.

## Results

Summary statistics

There were 64,6731 visits, including return visits, during the period 2006-2015. The estimated annual increment was 4,149/year, which started at 56,757 visits in 2006 and reached 71,289 visits in 2015. If this rate of increase continues at the same magnitude in 2030, the ED visit volume would be twice the number of visits in 2013. Table 1 shows that on average there were 177 visits/day, with the highest daily average at CTAS3 level visits and only 1-2 homeless visits per day. CTAS1 level visits were limited to summary statistics and were omitted for further trend analysis due to small numbers that showed sporadic patterns over time. Table 1 shows that the trends and patterns of homeless and non-homeless visits were statistically different with non-overlapping confidence intervals. 

**Table 1:** Halifax city daily visits patterns across demographic characteristics averaged over the period 2006-2015 CTAS = Canadian Trauma Acuity Scale, CI = confidence interval, CTAS1 = Canadian Trauma Acuity Level 1 (Resuscitation), CTAS2 = Canadian Trauma Acuity Level 2 (Emergent), CTAS3 = Canadian Trauma Acuity Level 3 (Urgent), CTAS4 = Canadian Trauma Acuity Level 4 (Semi-urgent), CTAS5 = Canadian Trauma Acuity Level 5 (Non-urgent)

	CTAS1	CTAS2	CTAS3	CTAS4	CTAS5
Non-homeless Average visits per day, (95% CI)					
Age group Less than 30	0.63 (0.61-0.65)	10.50 (10.16-10.84)	45.37 (43.90-46.84)	34.82 (33.70-36.0)	7.24 (7.01-7.48)
30-50 years	0.27 (0.26-0.28)	4.62 (4.47-4.77)	12.10 (11.70-12.49)	7.05 (6.82-7.28)	1.44 (1.39-1.49)
50 plus years	0.68 (0.66-0.70)	11.17 (10.81-11.53)	28.97 (28.03-29.91)	9.20 (8.90-9.50)	1.51 (1.46-1.56)
Gender Male	1.01 (0.98-1.04)	13.53 (13.09-13.97)	37.41 (36.20-38.62)	26.27 (25.42-27.12)	5.24 (5.07-5.41)
Female	0.57 (0.55-0.58)	12.76 (12.35-13.17)	49.02 (47.43-50.61)	24.80 (24.00-25.61)	4.96 (4.80-5.12)
Total (non-homeless)	1.57 (1.55-1.59)	26.03 (25.91-26.70)	86.45 (86.04-86.87)	51.10 (50.76-51.43)	10.24 (10.07-10.41)
Homeless Average visits per 10 days, (95% CI)					
Age group Less than 30	0.073 (0.071-0.076)	1.67 (1.61-1.72)	4.10 (3.96-4.22)	3.08 (2.98-3.18)	1.20 (1.16-1.24)
30-50 years	0.014 (0.013-0.014)	0.32 (0.31,0.33)	1.05 (1.02-1.09)	0.66 (0.64-0.69)	0.35 (0.34-0.36)
50 plus years	0.006 (0.005-0.006)	0.22 (0.21-0.23)	0.72 (0.70-0.74)	0.58 (0.56-0.60)	0.26 (0.25-0.27)
Gender Male	0.074 (0.071-0.076)	1.66 (1.60-1.71)	4.39 (0.42-0.45)	3.27 (3.17-3.37)	1.37 (1.32-1.41)
Female	0.019 (0.018-0.02)	0.55 (0.52-0.56)	1.47 (1.43-1.52)	1.06 (1.02-1.09)	0.44 (0.43-0.45)
Total (homeless)	0.093 (0.090-0.096)	2.3 (2.2-2.3)	5.9 (5.7-6.1)	4.3 (4.2-4.5)	1.8 (0.17-0.19)

Non-homeless EDVR trends

**Figure 1:**
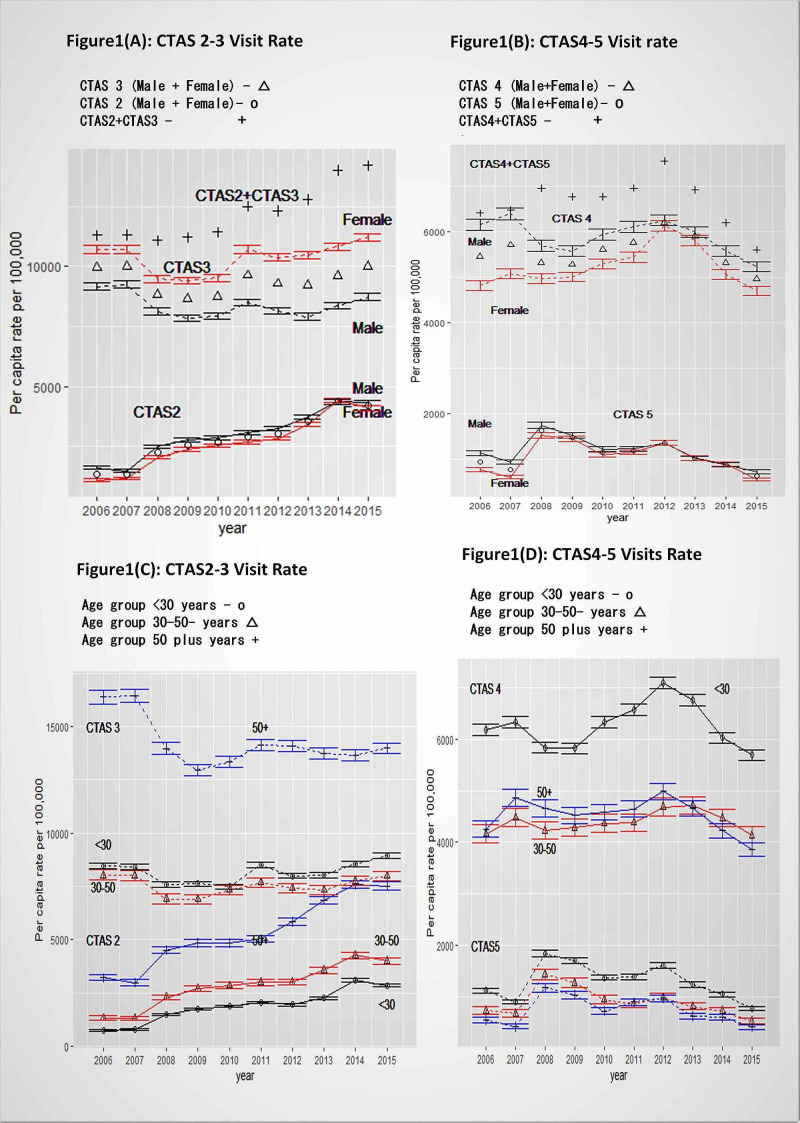
Halifax city non-homeless age-gender and CTAS specific annual per capita rates (per 100,000 population) with 95% confidence intervals CTAS = Canadian Trauma Acuity Scale

An annual linear per capita non-homeless EDVR increment trend was estimated as 328/year (CI:245-411, per 100,000) for all CTAS1-5 visits combined. Annual CTAS2 EDVR showed a statistically significantly linearly increasing trend, with 335/year (CI:280-391, per 100,000), and statistically similar increment of 351/year (CI:243-459) was shown after combining high acuity CTAS2-3 visits (Figure 1A). There was an annual linear declining trend of CTAS5 EDVR (121/year fewer visits, CI:79-163) during the eight-year period (2008-2015) and this decline alone did not influence overall CTAS2-4 EDVR increment for this period, which was 363/year (95% CI:236-490) (Figure 1B, 1D). But when an annual CTAS4 visits declining trend of 427/year (CI:350-503) for the four-year-period (2012-2015) was combined with the CTAS5 declining trend, then overall CTAS2-4 EDVR annual incremental trend can be flattened. 

Non-homeless Standardized Emergency Visit Rate (SEVR) trends

**Figure 2:**
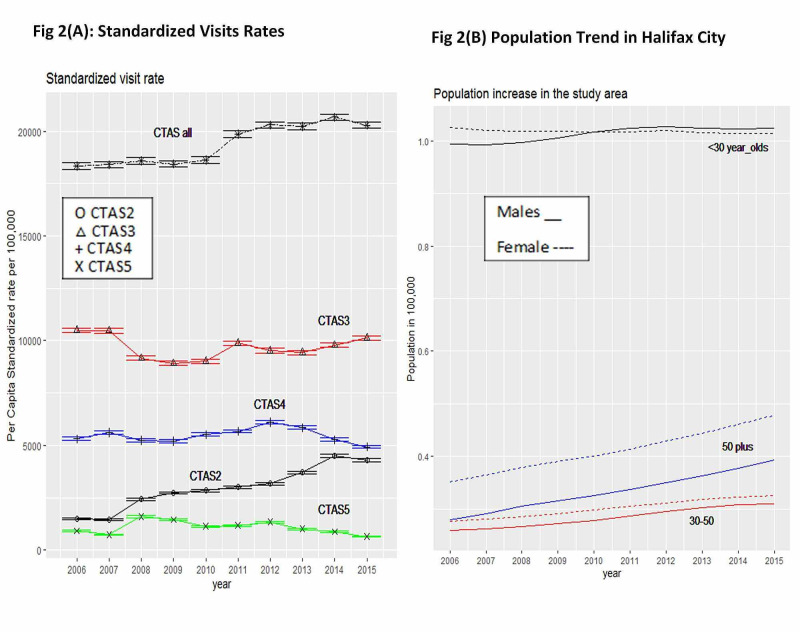
Halifax city non-homeless adult standardized visit rates with 95% confidence intervals (2A) and adult (>17 years) population time trends (2B) CTAS = Canadian Trauma Acuity Scale

In the study city of Halifax municipality, the population of adults older than 16 increased with a linear trend of 4,149/year (CI:4012-4287, per 100,000). The highest population increase was for those 50 years and older (2,604/year CI:2494-2714), followed by 30-50-year-old group (1,223/year, CI:1,138-1,309), and the lowest trend was for the 16-30 age group (322/year, CI:170-473) (Figure 2B). Figure 2A shows the SEVR trends after adjusting for Halifax municipality 2011, age-gender specific census data. The overall SEVR annually increased at a rate of 294/year (CI:207-382). In the order of magnitude, CTAS3 SEVR was the highest, followed by CTAS2, CTAS4, and CTAS5 (Figure 2A). CTAS2 SEVR continued to increase at a rate of 316/year (CI:261-372), also at a statistically similar rate incline trend to EDVR (Figure 2B and Figure 1A, 1D). During the study period, the annual SEVRs for CTAS3 were stable, but CTAS4 SEVR declined at a rate of 416/year (CI:338-494) for the four-year period of 2011-2015, and CTAS5 SEVR declined at a rate of 117/year (CI:75-158), statistically similar to non-standardized EDVR rate declining trend for CTAS4-5. 

Non-homeless trends by gender

The highest daily average visits were noted for female CTAS3 visits of 49/day (CI: 47.43-50.61) and were statistically significantly different for male CTAS3 visit average of 37/day (CI:36.20-28.62). The CTAS1 visits daily average was higher for males with one per day (CI: 0.98-1.04) than for females which was less than one per day (average 0.57/day, CI:0.55-0.58) (Table 1). Per capita annual CTAS2 EDVR incremental time trends were statistically similar for males and females (Figure 1A). Females showed statistically significantly higher CTAS3 EDVR compared to males throughout the study period (Figure 1A). Males had significantly higher CTAS4 EDVR (except in 2012) than females and there were no gender differences in CTAS5 EDVR (Figure 1B).

Non-homeless trends by age group

Patients aged 50 and over made the highest average daily CTAS1 and CTAS2 visits, while those 30 or less had the most CTAS4-5 visits (Table 1, Figure 1C, 1D). CTAS2-3 visit rates were higher for 50 and older age group (Figure 1C) and CTAS4-5 rates were notably higher for those younger than 30 in each year (Figure 1D). There was a linearly increasing annual CTAS2 EDVR time trend for all three age groups. Age of 50 and older group showed the highest annual incremental CTAS2 trend at the rate of 521/year (CI: 433-608), followed by 30-50-year-olds at 316/year (CI 255-377), and the <30-year-olds at 242/year (CI: 180-303) (Figure 1C). The low-acuity CTAS5 rates showed statistically similar annual declining linear trends across all age groups (Figure 1D). 

Homeless EDVR trends

On average, there were one to two homeless visits/day, and younger homeless patients had more average visits/day than older ones across all CTAS levels. The daily visit average peaked at CTAS3 level visits (Table 1). Homeless men had more average visits/day in all CTAS levels than homeless women. Homeless EDVR annual incremental trend was 478/year (CI:359-596, per 100,000) and no statistically significant gender differences were noted. CTAS2 EDVR by homeless patients linearly increased by 219/year (CI:193-246), and this trend was more prominent for males (247/year, CI:207-287) than for females (155/year, CI:82-228), with no statistical differences. Only the age group <30-year-old showed a CTAS2 annual EDVR increasing trend (643/year, CI:396-890). CTAS3 homeless EDVR also increased annually by 182/year (CI:99-264), accounted by homeless males (235/year, CI:116-353), less than 30-year-olds (227/year, CI:77-377), and above-50-year-olds (287/year, CI:86-488). 

## Discussion

Acuity level-based per capita annual ED visit rate trends for a decade-long period has never been studied in Canada. No study in Canada has documented the possibility of flattening the escalating ED visit trends. We noted that this can be achieved by maintaining an annual declining trend in low-acuity-level visits at a particular rate. The Canadian Foundation for Healthcare Improvement (CFHI) reported an annual ED visit increment of 40% for Canada, estimated as from 15 million per year in 2013 (observed) to over 21 million in 2043 (projected) [4]. This estimate was statistically similar to the Alberta ED visit annual increments shown for a shorter period of 2013-2015 [4]. The findings of our study also showed statistically similar estimated percentage increment of 49% (CI:37-62%), from the 69,163 ED visits reported in the year 2013, which would increase up to 103,282 in 2043, if the 10-year annual estimated trend of 328/year (per 100,000) continues at the same rate. Most importantly, if the low-acuity-level declining rate within the four-year period (2012-2015) can be maintained, the incremental trends in the percentage and, more accurately, in the per capita rate will be nullified. 

The decade-long trend analysis across acuity levels allowed us to capture the time segments where the trend changed the direction from incline to decline in the low-acuity level (semi-urgent and non-urgent) ED visit rates within the last four-year study period of 2012-2015. Relying on this change, we demonstrated that if these declining rates could be maintained at the same magnitude and direction, the overall ED visit increment would become stable. We emphasize the importance of examination of time segments of lengthy records of data to capture changes in the directions of annual trends across acuity levels and which enables to identify the ecological relationship with healthcare service delivery changes that happened during those time segments. The Professional Standard on the Standard of Care for Walk-in Clinics was approved during the period of 2010-2015. Primary care service delivery has been improved after introducing walk-in clinics operating for long hours, including weekends, and these changes can only be captured after examining specific time-segments of a lengthy record of acuity level-based ED visit data. 

Canadian studies that examined acuity level-based annual trends have combined CTAS1-3 (resuscitation, emergent, urgent) levels as high acuity. For the two provinces - Ontario and British Columbia - high-acuity-level ED visit percentage increments for the period 2008-2012 were 2.6% and 14.9% per year [2,14]. Both provinces attract the most immigrants to the country and these annual incremental trends did not account for the year-to-year variation in the census figures either by estimating per capita rates or standardized rates. Our census-unadjusted ED increment for the period 2008-2012 was 4.7% (CI: 3.04-6.30), greater than Ontario and lesser than British Columbia. Out study data also showed a decreasing trend for CTAS5-level visits, a direction that was followed in Ontario and British Columbia data for the same period [15]. It is unclear whether the differences noted in different geographies are due to changes in census figures or due to the increase of the prevalence of high-acuity-level illnesses. Per capita visit rates allow identification of annual trends adjusting for annual changes in population census and the standardized visit rates allows further comparisons across geographic locations. 

We found females as having higher CTAS3 EDVR than males and males having higher CTAS4 EDVR than females. However, there were no gender differences in ED visits across CTAS found for a sample of patients followed by a family health team in Toronto [14]. We need further research to understand whether males have less family doctor follow-ups than females to understand the gender discrepancy in semi-urgent (CTAS4) visits. There are nearly 15,000 people, that is, 12.7% of the study city of Halifax population, without a family physician [3].

Our study finding of homeless having higher EDVR than non-homeless was consistent with other Canadian studies [16]. We found young (less than 30 years of age) homeless males as having the highest high-acuity-level visit increase. There are no CTAS-specific homeless ED use findings available in Canadian literature; future research studies on this population should consider acuity level-based analysis to assist with future homeless healthcare planning aiming at young male homeless populations. 

The strength of data lies in the large volume of data collected over a 10-year period, analyzed by acuity level of presentations, identifying the direction of trend changes over time and adjusting with standardized population census data that allows comparison across populations. Limitations for generalizability exist since the findings were drawn from a single site in a small province. Though most of our findings were consistent with other Canadian city ED study findings, these ecological study findings need to be confirmed with larger national studies with more recent ED visit data that includes more details of the acuity level categorization, including clinical presentations to ED. 

Expanded national multicity research on acuity-based visits, with presenting health conditions, should confirm whether these acuity level- and demographic group-specific time trends research findings presented in this paper can be generalized to the Canadian population. Demographic group-specific ED visit patterns across acuity levels need to be taken into consideration in future research. 

## Conclusions

This study revealed a trend of increasing high-acuity-level CTAS2 ED visits at a higher rate than other acuity level-based visits. There was a decreasing time trend in annual rates of low-acuity-level semi-urgent and non-urgent visits within the four-year period of 2012-2015, and if this magnitude of trend can be maintained, the overall ED visit rate annual increment could become stable. This study results provided details of demographic populations contributing to high acuity level increasing trends. Both males and females over the age of 50 contributed to the Halifax high acuity level (CTAS2) increasing time trend, one that remains unchanged even after adjusting for annual population increase. The annual increase in CTAS2 EDVR, if it continues at the same rate, would have a significant impact on ED service planning, and this burden can be alleviated if declining low-acuity-level visit trends were not maintained at the estimated rate noted in this paper. The acuity level-based trends across demographic characteristics noted in this paper provide potential areas to target clinical investigations to examine trends in presenting health conditions to help healthcare improvement for demographically vulnerable populations. 
